# A systematic review of Streptococcus Mutans and Veillonellae species interactions in dental caries progression: Positive or Negative impact?

**DOI:** 10.12688/f1000research.155987.1

**Published:** 2024-09-20

**Authors:** Faizul Hasan, Hendrik Setia Budi, Rajesh Ramasamy, Tantiana Tantiana, Rini Devijanti Ridwan, Ervina Restiwulan Winoto, Prawati Nuraini, Juni Handajani, Ariadna Adisattya Djais, Silvia Anitasari

**Affiliations:** 1 Faculty of Nursing, Chulalongkorn University, Bangkok, 10330, Thailand; 2 Department of Oral Biology, Dental Pharmacology, Faculty of Dental Medicine, Universitas Airlangga, Surabaya, East Java, 60132, Indonesia; 3 Immunology Unit, Department of Pathology, Faculty of Medicine and Health Sciences, Universiti Putra Malaysia, Serdang, Selangor, 43400, Malaysia; 4 Department of Oral Biology, Faculty of Dental Medicine, Universitas Airlangga, Surabaya, East Java, 60132, Indonesia; 5 Department of Orthodontics, Faculty of Dental Medicine, Universitas Airlangga, Surabaya, East Java, 60132, Indonesia; 6 Department of Pediatric Dentistry, Faculty of Dental Medicine, Universitas Airlangga, Surabaya, East Java, 60132, Indonesia; 7 Department of Oral Biology, Faculty of Dentistry, Universitas Gadjah Mada, Yogyakarta, Special Region of Yogyakarta, 55281, Indonesia; 8 Department of Oral Biology, Faculty of Dentistry, Universitas Indonesia, Jakarta, 10430, Indonesia; 9 Department Medical Microbiology, Medical Program, Faculty of Medicine, Universitas Mulawarman, Samarinda, East Kalimantan, 75119, Indonesia

**Keywords:** dental caries, Streptococcus mutans, Veillonellae species, lactic acid, substrates competition

## Abstract

**Background:**

The interaction between *Streptococcus mutans (S. mutans)* and *Veillonella species* ( *Veillonella spp.*) is unclear. This study aims to investigate the interaction between *S. mutans* and *Veillonella spp.* on caries development using systematic review.

**Methods:**

This systematic review was accorded to the guideline of the Preferred Reporting Items for Systematic Reviews and Meta-Analyses. Three electronic databases, namely PubMed, Embase, and the Cochrane library, were used to conduct a systematic search for eligible studies from their inception until July 18, 2023. PROSPERO registration number was No. CRD42023445968.

**Results:**

We initially identified 4,774 articles. After eliminating duplicates and irrelevant articles, 11 studies met the inclusion criteria. The studies revealed important aspects of the relationship between *S. mutans* and *Veillonellae spp.* in dental caries. One significant finding is that *Veillonellae spp.* can affect the acid production capacity of *S. mutans.* Some studies indicate that *Veillonellae spp.* can inhibit the acid production by *S. mutans*, potentially reducing the cariogenic process. Another aspect is the competition for substrates. *Veillonellae spp.* utilize lactic acid, which is a by product of *S. mutans* metabolism, as a source of carbon. This metabolic interaction may decrease the availability of lactic acid for *S. mutans*, potentially influencing its cariogenic potential.

**Conclusions:**

This systematic review highlights the emerging evidence on the interaction between *S. mutans* and *Veillonellae spp.* in dental caries. The findings suggest that *Veillonellae spp.* can modulate the acid production, and substrate competition of *S. mutans*, potentially influencing the cariogenic process.

## Introduction

The oral cavity is a habitat that provides a diversity of microbial species, and it is estimated that more than 700 species of bacteria are present in the oral cavity. ^1^ The teeth, tongue, cheeks, gingival sulcus, tonsils, and palate provide a favourable environment for microorganisms to thrive. The oral cavity’s surface is coated with many bacteria in dental plaque, known as a dental biofilm. ^2^^,^^3^ The main oral bacteria are gram-positive and gram-negative, followed by aerobic and anaerobic. The commensal flora works in harmony with the host, but this relationship can become disharmonious due to changes in the microenvironment (microenvironment), resulting in disease states. Predominant oral diseases such as caries and periodontal disease are caused by microflora that are not in line with the host (dysbiosis). ^4^ The role of beneficial bacteria is to prevent invasion of pathogenic bacteria. ^1^ Polymicrobial community in oral biofilms include *Streptococcus, Actinomyces, Lactobacillus, Veillonellae, Neisseria,* and *Eubacterium.* ^5^ This biofilm can thrive in homeostasis with a human host but is also the cause of the two most common human diseases, dental caries and periodontitis. ^6^^,^^7^

*Streptococcus mutans* ( *S. mutans*) is a normal flora in the oral cavity, including gram-positive facultative anaerobes. In certain circumstances, these bacteria can turn into pathogens due to predisposing factors, namely oral hygiene. ^8^ Dental caries is caused by multifactorial, one of which is food left on the surface or between the teeth. Sweet food with high glucose, such as carbohydrates, candy, sugar and other sweet foods, will form a layer of plaque (dental biofilm) on the tooth surface as a colony for a multi microorganism. ^9^ Dental caries and plaque are among the most common diseases worldwide and are caused by a mixture of microorganisms and food. ^10^ Acid-producing bacteria, especially *S. mutans*, will colonize the tooth surface and cause damage to the hard tooth structure due to the fermentation of carbohydrates into sucrose and fructose. ^11^ Lactic acid formed by *S. mutans* bacteria in plaque will cause demineralization of tooth enamel and decrease saliva acidity (pH), resulting in caries over time. ^12^

However, lactic acid is a source of life for other bacteria in the vicinity, such as *Veillonellae species* ( *Veillonellae spp.*) and is closely related to lactic acid-producing bacteria. ^13^^,^^14^ Bacteria in the oral cavity will reduce high concentrations of nitrate in saliva to nitrite, and nitric oxide. ^15^ *Veillonela spp.* is a gram-negative anaerobic bacteria that can convert lactic acid into weak nitric acid (NO _3_^-^), which is then reduced to produce nitrite (NO _2_^-^), and nitric oxide (NO) which is a source of defence in the oral cavity against pathogenic bacteria and raises the pH of the oral cavity. ^16^^,^^17^ This process is known as the nitrate-nitrite-NO pathway. ^18^ The biological activity of nitrite is lower than that of NO, while nitrate has no biological activity. The half-life of NO is very short, so it only has activity around its biosynthetic site. ^19^

The symbiosis between bacteria can certainly benefit or harm the host. *Veillonella spp.* may reduce lactic acid that causes dental caries produced by *S. mutans.* However, there is a lack of pooled evidence investigating the interaction between *S. mutans* and *Veillonella spp.* on caries development using systematic review. Therefore, this systematic review examined the interaction between *S. mutans* and *Veillonella spp.* on caries development.

## Methods

### Data sources and searches

This systematic review was accorded to the guideline of the Preferred Reporting Items for Systematic Reviews and Meta-Analyses. ^20^ The study registration number was CRD42023445968 on the International Prospective Register of Systematic Reviews website. Three electronic databases, namely PubMed, Embase, and the Cochrane library, were used to conduct a systematic search for eligible studies from their inception until July 18, 2023. We used the following keyword combinations to search for articles: (“Streptococcus mutans”) OR (“S. mutans”) AND (Veillonella) OR (“Veillonella spp”) AND (“Dental Caries”) OR (“Tooth Decay”), the searching strategies were listed in Table 1. In addition, the reference lists of the eligible articles were manually searched to identify additional relevant publications.

**Table 1.** Search strategy.

Database	Keywords
PubMed	((((“Streptococcus mutans”[All Fields] OR “s mutans”[All Fields]) AND (“veillonella”[MeSH Terms] OR “veillonella”[All Fields] OR “veillonellae”[All Fields])) OR “Veillonella spp”[All Fields]) AND “Dental Caries”[All Fields]) OR “Tooth Decay”[All Fields]
EMBASE	(“Streptococcus mutans”) OR (“S. mutans”) AND (Veillonella) OR (“Veillonella spp”) AND (“Dental Caries”) OR (“Tooth Decay”)
Cochrane Library	(“Streptococcus mutans”) OR (“S. mutans”) AND (Veillonella) OR (“Veillonella spp”) AND (“Dental Caries”) OR (“Tooth Decay”)

### Study selection

We included full-text studies that fulfilled the following criteria: (1) individuals with dental caries, early childhood caries (ECC), or root caries, compared or not with a control group without caries; (2) reported the interaction between Streptococcus mutam and Veillonella in dental caries; and (3) the study employed a cross-sectional, case–control, prospective, retrospective, or randomized controlled. We defined the dental caries by Decay-missing-filled teeth index (DMFT) or other standard instrument used to define the dental caries. There were no language or publication date restrictions.

Two investigators (H.S.B. and F.H.) searched the electronic databases independently and screened and reviewed the qualified publications. The third investigator (R.D.R.) assisted in resolving any discrepancies through discussion.

### Quality assessment

**Study outcome**

To assess the methodological quality of the included studies, we used the **Joanna** . ^21^ We scored each item as “yes,” “no,” “unclear,” or “not applicable”.

**Data extraction and synthesis**

Two reviewers (H.S.B. and F.H.) extracted data and evaluated the included studies independently. Any differences were resolved through discussion. We extracted the first author’s name, publication year, study location, study design, sample demographics, sample size, and the interaction betweem Steptococcus mutan and Veillonella in dental caries. All data were presented in tables.

## Results

### Selection, inclusion, and characteristics of studies

**Figure 1.**
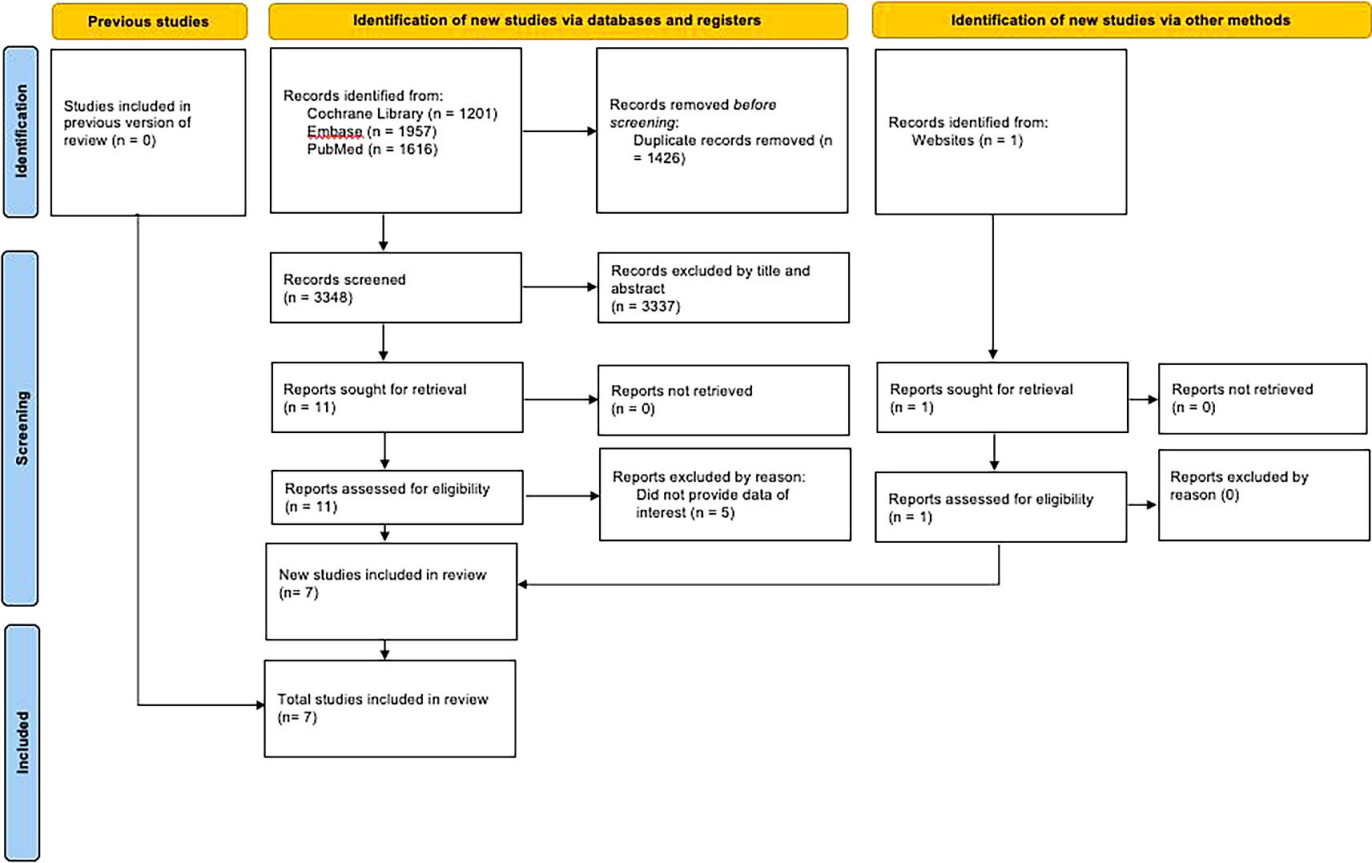
PRISMA flow diagram.

As presented in Figure 1, we initially identified 4,774 articles. After eliminating duplicates and irrelevant articles, 11 studies met the inclusion criteria. We added 1 studies from other sources. Five studies were excluded by reason were presented in Table 2. In total, there were 7 studies were included for the systematic review.

**Table 2.** List of the excluded studies after a full-text review.

Author, year	Reasons of exclusion
Do, 2015	Did not provide data of interest
Mashima, 2018	Did not provide data of interest
Rocas, 2015	Did not provide data of interest
Shen, 2005	Did not provide data of interest
Wang, 2017	Did not provide data of interest

Among the included studies, there were 408 participants in total. The age was ranges from 1 to 3.89 years old. Five studies were conducted in United State of America and the two studies were in Hong Kong and Indonesia. Other detail of participant characteristics were presented in Table 3.

**Table 3.** Participant characteristic.

Author, year	Country	Sample size		Age		Male		Female		Inclusion and exclusion criteria
		Caries	Control	Caries	Control	Caries	Control	Caries	Control	
Agnello, 2017	USA	30	20	42.8±12.2 mo	37.4±10.3 mo	19	9	11	11	***Inclusion:*** Children who were <72 months of age and identified by their parent or legal caregiver as being Canadian First Nations or Métis. ***Exclusion:*** Children who had taken antibiotics within the last 3 months.
Chalmers, 2015	USA	9	NA	3.89 yr	NA	6	NA	3	NA	***Inclusion:*** The child was medically healthy, had not used antibiotics within the last 3 months, and the parent or guardian was willing to consent to the child’s clinical examination and microbial sampling. ***Exclusion:*** NA
Gross, 2012	USA	36	36	23.6 (12-36) mo		46%		54%		***Inclusion:*** Subjects with dental caries and a dentally healthy control group were recruited. For the caries group was the presence of at least two maxillary incisors with white spot lesions, no cavitated lesion greater than 1 mm, and no existing restorations ***Exclusion:*** Age greater than 36 months, indications for infective endocarditis prophylaxis, and professional cleaning in the past 30 days.
Jiang, 2016	Hong Kong	20	20	3.3 yr	3.35 yr	10	10	10	10	***Inclusion:*** Chinese children aged 3–4 years old ***Exclusion:*** Had a serious health condition, were under regular medication, refused to cooperate in dental examination or saliva collection, or were absent from school on the examination day.
Setiawan, 2020	Indonesia	62	25	2-3 yr		42		45		***Inclusion:*** A child should be in good health, have no systemic abnormalities, and be willing to have an oral examination ***Exclusion:*** Children who had an abnormality in their mouth, such that they could not open their mouths or have been taking antibiotics for a long time.
Tanner, 2011	USA	42	40	3.97±0.12 yr	3.68±0.16 yr	24	21	18	19	***Inclusion:*** The child was medically healthy and had not used antibiotics within the last 3 months and the parent or guardian was willing to consent to the child’s clinical examination and microbial sampling. ***Exclusion:*** NA
Milnes, 1985	USA	5	4	10-16 mo		NA	NA	NA	NA	***Inclusion:*** Canadian Indian children with age 10-16 months. ***Exclusion:*** NA

mo= months; NA= not applicable; USA= United State of America; yr= years.

Table 4 depicted the studies characteristic. For the caries type, there were 4 studies were ECC type, and 3 studies were severe ECC. The definition to detect caries criteria was using DMFT, DMFS, white spot, gingivital index, and caries dentition criteria.

**Table 4.** Study characteristics.

Author, year	Instrument	Caries type	Results
Agnello, 2017	DMFT	S-ECC	The Relative abundance (%) median (range) of *Veillonella* dispar in caries subject was 3.0 (0.33 to 19.0) and for the control was 2.2 (0.15 to 9.4). For *Streptococcus mutans* was 0.73 (0.02 to 22.9) in caries group and 0.15 (0.006 to 10.4) in control group.
Chalmers, 2015	Extensive caries in the primary dentition	S-ECC	There were significant different (P=0.033) of relative abundance mean percentage and standard deviation of *Veillonella* in caries subject was 5.25 (±6.65) and for control was 15.22 (±9.48). For *Streptococcus* was 35.63 (±17.04) for caries subject and 32.11 (±18.91) for control group.
Gross, 2012	white spot or cavitated lesion	ECC	Veillonella, which metabolizes lactate, was associated with caries and was highly correlated with total acid producing species. Among children without previous history of caries, *Veillonella*, but not *S. mutans* or other acid-producing species, predicted future caries. There was significant correlation (r ^2^= 0.38) between relative levels of *Veillonella* and acidogenic streptococci in white spot lesions. The total % abundance of *S. mutans*, *S. sobrinus*, and *S. vestibularis/salivarius* combined is plotted against the abundance of the Veillonella atypica/dispar/parvula group expressed as a fraction of the remaining community.
Jiang, 2016	DMFT	ECC	At the genus level, the caries-affected group contained 117 genera, while the caries-free group contained 123 genera. *Streptococcus*, *Prevotella*, *Veillonella*, *Neisseria*, *Rothia*, *Haemophilus*, and *Gemella* constituted 75% of the caries-affected salivary microbial communities and 74% of the caries-free salivary microbial communities, respectively. According to the current study, salivary "caries-associated" species (such as *R. dentocariosa*, *A. graevenitzii*, and *F. periodonticum*) may be potential biomarkers for screening and assessing caries risk in children.
Setiawan, 2020	DMFS	ECC	The average proportion of *Veillonella* spp. in caries-free children (2.13±2.30) was lower than in ECC children (3.29±6.83), suggesting that *Veillonella* spp. may be a risk factor for ECC.
Tanner, 2011	plaque and gingival indexes, and gingival bleeding	S-ECC	Despite not being acidogenic, *Veillonella* species are frequently linked to caries. By promoting the proliferation or survival of cariogenic species, as has been shown for the interactions between *Veillonella* species and *S. mutans*, they may play a crucial role in the caries biofilm.
Milnes, 1985	White spot lesion and cavitation	ECC	*Streptococcus mutans*, *Lactobacillus*, and *Veillonella* levels differ significantly between caries and caries-free subjects. Veillonella were discovered in substantially greater numbers on the caries subject. The discovery was unrelated to the development of a lesion and may reflect an increase in lactic acid in plaque. Veillonella modifying the carries attack does not appear likely.

DMFT = The Decayed, Missing, and Filled Teeth; DMFS = The Decayed, Missing, and Filled Surface; ECC = early children caries; S-ECC = severe early children caries.

### Interaction between *Streptococcus mutans* and *Veillonellae species* on dental caries

The studies revealed important aspects of the relationship between *S. mutans* and *Veillonellae spp.* in dental caries. One significant finding is that *Veillonellae spp.* can affect the acid production capacity of *S. mutans.* Some studies indicate that *Veillonellae spp.* can inhibit the acid production by *S. mutans*, potentially reducing the cariogenic process.

Another aspect is the competition for substrates. *Veillonellae spp.* utilize lactic acid, which is a by product of *S. mutans* metabolism, as a source of carbon. This metabolic interaction may decrease the availability of lactic acid for *S. mutans*, potentially influencing its cariogenic potential.

Limited evidence suggests that *Veillonellae spp.* might have an impact on the host immune response and modulate the inflammatory processes associated with dental caries. Further research is necessary to understand the specific mechanisms involved in these interactions.

### Meta-analysis

The meta-analysis cannot be performed due to the limitation of the number of included studies.

### Methodological quality of included studies

Most studies used a cross-sectional study design. Of these, 4 studies did not state their strategies for addressing confounding factors. All studies measure outcomes in a valid and reliable manner. Other details regarding the methodological quality of studies are summarized in Table 5.

**Table 5.** Risk of methodological bias score of the studies.

Authors, year	1	2	3	4	5	6	7	8
Agnello, 2017	Y	Y	Y	Y	Y	Y	Y	Y
Chalmers, 2015	Y	Y	Y	Y	Y	NA	Y	Y
Gross, 2012	Y	Y	Y	Y	Y	NA	Y	Y
Jiang, 2016	Y	Y	Y	Y	Y	NA	Y	Y
Setiawan, 2020	Y	Y	Y	Y	Y	Y	Y	Y
Tanner, 2011	Y	Y	Y	Y	Y	Y	Y	Y
Milnes, 1985	Y	Y	Y	Y	NA	NA	Y	Y

**1**, Were the criteria for inclusion in sample clearly defined? **2**, Were the study subjects and the setting described in detail? **3**, Was the exposure measured in a valid and reliable way? **4**, Were objective, standard criteria used for measurement of the conduction? **5**, Were confounding factors identified? **6**, Were strategies to deal with confounding factors stated? **7**, Were the outcomes measured in a valid and reliable way? **8**, Was appropriate statistical analysis used? N, no; NA, not applicable; UC, unclear; Y, yes.

## Discussion

This systematic review highlights novel findings about the interaction of *Veillonellae spp.* and *S. mutans* in dental caries. The results imply that *Veillonellae spp.* can control the substrate competition and acid production of *S. mutans*, potentially affecting the cariogenic process. This interaction may also be influenced by host-microbe interactions. Because our study implied rigorous methodology, hence, the finding should be highly considered.

*Streptococcus mutans* is a bacterium commonly found in the human oral cavity and is known for its role in dental caries formation. ^22^ However, recent studies have shown that *Veillonellae*, anaerobic bacteria commonly found in the oral cavity, play a role in inhibiting this caries progression. ^23^ *Veillonellae*, on the other hand, are anaerobic bacteria that thrive in the same environment. Studies have shown that these two bacteria have a mutually beneficial relationship. ^24^

*Streptococcus mutans* is known for its ability to ferment dietary carbohydrates, producing acids as by products. These acids can lead to the demineralization of tooth enamel, increasing the risk of dental caries. ^25^ However, Veillonellae have been found to metabolize the lactate produced by *S. mutans.* ^26^ Lactate is a key component of the acid production process. ^27^ By lowering the acidity, *Veillonellae* contribute to maintaining a more balanced pH level, ^28^ which is crucial for oral health and prevents enamel demineralization. ^29^

Furthermore, the presence of *Veillonellae* has been shown to inhibit the growth and colonization of other potentially harmful bacteria in the oral cavity. This further emphasizes the beneficial role of *Veillonellae* in maintaining oral health. The exact mechanisms by which Veillonellae inhibit the growth of these harmful bacteria are still being studied, but it is believed that they compete for resources or produce antimicrobial substances. The symbiosis between *S. mutans* and *Veillonellae* plays a significant role in maintaining the homeostasis of the oral microbiome. ^30^^,^^31^

The relationship between *Veillonella spp.* abundance and dental caries (specifically *Early Childhood Caries or ECC*) is not entirely consistent across all studies. While some studies have reported lower levels of Veillonella in caries-free individuals compared to those with ECC, other studies have reported different findings. In some research, lower levels of *Veillonella spp.* have been associated with a higher risk of dental caries, including ECC. ^32^ This could be because *Veillonella*, as an acid-resistant bacteria, plays a role in modulating the oral environment and reducing acidity, which is beneficial in preventing dental caries. On the other hand, other studies have shown contrasting results, reporting either higher levels of Veillonella or no significant difference in its abundance between caries-free children and those with ECC. ^33^

Some studies have shown a higher abundance of *Veillonella* in caries subjects compared to control subjects, suggesting a potential role of *Veillonella* in caries progression. On the other hand, other studies have found no significant difference in *Veillonella* levels between the two groups. The difference in *Veillonella* abundance between caries subjects and controls could be attributed to several factors related to the oral microbiome and the development of dental caries. The oral microbiome is a complex ecosystem consisting of various bacterial species, and its composition can be influenced by multiple factors, including diet, oral hygiene practices, genetics, and environmental factors. ^34^

The potential reasons for the difference in *Veillonella* amount between caries subjects and controls are diet, oral hygiene practices, acidity (pH), host genetics and immune and disease progression response. The type and frequency of dietary sugar intake can influence the abundance of different bacterial species in the oral cavity. High sugar consumption provides more food for acid-producing bacteria like *S. mutans*, which can lead to a shift in the microbial balance. ^35^ As *Veillonella* can utilize metabolic by products of acid-producing bacteria, its abundance may change in response to variations in sugar consumption. Regular and effective oral hygiene practices, such as brushing and flossing, can help maintain a healthier oral microbiome by reducing the accumulation of plaque and the growth of cariogenic (caries-causing) bacteria. Inadequate oral hygiene may promote the growth of cariogenic bacteria at the expense of acid-resistant bacteria like *Veillonella.* Dental caries development is associated with a decrease in oral pH due to acid production from certain bacteria. *Veillonella* is known to thrive in low-pH environments and can help modulate the acidity in the oral cavity. Consequently, its abundance may be affected by changes in pH levels caused by the presence of cariogenic bacteria. ^10^ Individual variations in host genetics and immune responses can influence the composition of the oral microbiome and susceptibility to dental caries. ^36^ The interactions between the host and specific bacterial species, including *Veillonella*, might impact its abundance in the oral cavity. Disease progression, dental caries is a dynamic process. As the disease progresses, the oral environment changes, which can lead to shifts in the composition of the oral microbiome, including *Veillonella.*

To understand the specific reasons for the difference in Veillonella abundance between caries subjects and controls, need to conduct comprehensive studies that take into multiple factors and potentially control for confounding variables. It is important to note that the conflicting results may be due to various factors, such as differences in study design, sample size, and methodology. Additionally, the oral microbiome is highly complex, and the interaction between different microorganisms may also influence the progression of caries. Further research is needed to fully elucidate the specific interactions and mechanisms involved in this symbiotic relationship between *S. mutans* and *Veillonellae.* Such knowledge could potentially lead to the development of targeted therapies or probiotics that promote the growth of *Veillonellae* to prevent or slow down the progression of dental caries.

## Conclusion

This systematic review highlights the emerging evidence on the interaction between *S. mutans* and *Veillonellae species* in dental caries. The findings suggest that *Veillonellae spp.* can modulate the acid production, and substrate competition of *S. mutans*, potentially influencing the cariogenic process. Additionally, host-microbe interactions may play a role in this interaction. Further research, including well-designed in vitro and in vivo studies, is needed to fully understand the mechanisms underlying this interaction. The knowledge gained from such investigations could pave the way for novel preventive and therapeutic strategies targeting the interaction between *S. mutans* and *Veillonellae* species to manage dental caries effectively.

## Ethics and consent

Ethical approval and consent were not required.

## Data Availability

No data are associated with this article. PRISMA checklist https://doi.org/10.5281/zenodo.11213481 Data are available under the terms of the Creative Commons Zero “No rights reserved” data waiver (CC0 1.0 Public domain dedication). Joanna Briggs Institute Critical Appraisal Tools can be assessed here: https://jbi.global/critical-appraisal-tools
