# Supplementary material for: A systematic review of Streptococcus Mutans and Veillonellae species interactions in dental caries progression: Positive or Negative impact?
Source: F1000Res. 2025 Jan 23;13:1080. Originally published 2024 Sep 20. [Version 2] doi: 10.12688/f1000research.155987.2 (PMC11757920; doi:10.12688/f1000research.155987.2)
Supplement: Peer review history

# Reviewer report: Reviewer response for version 2

**DOI:** 10.5256/f1000research.176959.r365051

**Published:** 2025-02-07

**Authors:** Tri Purnama Dewi

**Affiliations:** 1 Universitas Mahasaraswati Denpasar, Denpasar, Bali, Indonesia

**Your Report**

**1. **In the "introduction" section, there is the word (microenvironment) in Parenthesis, does it indicate additional information?. 

 In the sentence "Dental caries and plaque are among the most common diseases worldwide and are caused by a mixture of microorganisms and food": there are several perceptions, whether plaque is also the most common disease, or the cause of dental caries consisting of a mixture of microorganisms

 2. For the "discussion" and "conclusion" sections, it would be better to be more organized/sequential in explaining "the relationship" which causes "the interaction" between the two species.

Are the rationale for, and objectives of, the Systematic Review clearly stated?

Yes

Is the statistical analysis and its interpretation appropriate?

Not applicable

If this is a Living Systematic Review, is the ‘living’ method appropriate and is the search schedule clearly defined and justified? (‘Living Systematic Review’ or a variation of this term should be included in the title.)

Yes

Are sufficient details of the methods and analysis provided to allow replication by others?

Yes

Are the conclusions drawn adequately supported by the results presented in the review?

Yes

Reviewer Expertise:

NA

I confirm that I have read this submission and believe that I have an appropriate level of expertise to confirm that it is of an acceptable scientific standard.

# Reviewer report: Reviewer response for version 2

**DOI:** 10.5256/f1000research.176959.r362027

**Published:** 2025-01-30

**Authors:** Atik Kurniawati, Sari Setyaningsih, Yohana Maria Penga

**Affiliations:** 1 Universitas Jember, Jember, East Java, Indonesia; 2 Biomedicine, Universitas Jember, Jember, East Java, Indonesia; 3 Oral Biology and Biomaterial, Universitas Jember, Jember, East Java, Indonesia

We have no further comments on the manuscript. The author has revised it as suggested by the reviewer.

Are the rationale for, and objectives of, the Systematic Review clearly stated?

Yes

Is the statistical analysis and its interpretation appropriate?

I cannot comment. A qualified statistician is required.

If this is a Living Systematic Review, is the ‘living’ method appropriate and is the search schedule clearly defined and justified? (‘Living Systematic Review’ or a variation of this term should be included in the title.)

Yes

Are sufficient details of the methods and analysis provided to allow replication by others?

Partly

Are the conclusions drawn adequately supported by the results presented in the review?

Partly

Reviewer Expertise:

Oral Microbiology, Oral Biology

We confirm that we have read this submission and believe that we have an appropriate level of expertise to confirm that it is of an acceptable scientific standard.

# Reviewer report: Reviewer response for version 2

**DOI:** 10.5256/f1000research.176959.r362025

**Published:** 2025-01-24

**Authors:** Zahreni Hamzah

**Affiliations:** 1 Department of Biomedical Science, Faculty of Dentistry, University of Jember, Jember, Indonesia

I have seen the revisions made, and I fully approve for indexing.

Are the rationale for, and objectives of, the Systematic Review clearly stated?

Yes

Is the statistical analysis and its interpretation appropriate?

Yes

If this is a Living Systematic Review, is the ‘living’ method appropriate and is the search schedule clearly defined and justified? (‘Living Systematic Review’ or a variation of this term should be included in the title.)

Yes

Are sufficient details of the methods and analysis provided to allow replication by others?

Yes

Are the conclusions drawn adequately supported by the results presented in the review?

Yes

Reviewer Expertise:

NA

I confirm that I have read this submission and believe that I have an appropriate level of expertise to confirm that it is of an acceptable scientific standard.

# Reviewer report: Reviewer response for version 1

**DOI:** 10.5256/f1000research.171233.r350940

**Published:** 2025-01-23

**Authors:** Atik Kurniawati, Yohana Maria Penga, Sari Setyaningsih

**Affiliations:** 1 Universitas Jember, Jember, East Java, Indonesia; 2 Oral Biology and Biomaterial, Universitas Jember, Jember, East Java, Indonesia; 3 Biomedicine, Universitas Jember, Jember, East Java, Indonesia

The urgency of this study is not yet clear. It is not mentioned that Veillonella sp. as a gram-negative anaerobic bacteria is mainly found in the gingival crevicular and tongue, which cannot use carbohydrates as an energy source. Instead, Veillonella sp. oxidizes lactic acid, which is the final metabolic product of bacteria that metabolize carbohydrates (including S. mutans), as a result the pathogenic potential of these bacteria in caries is reduced. So by manipulating part of this ecosystem, the pathogenic potential of bacteria is reduced or perhaps eliminated.

 Methods : Did it include exclusion criteria such as: 1) review article, 2) non-human study?

 It is necessary to add a table of Conclusions on the interaction between S. mutans and Veillonellae sp. on caries, as positive or negative categories

 It is necessary to discuss, from most of the reference results, whether they show the interaction of S. mutans and Veillonell sp. on caries as positive or negative. It is also necessary to explain the limitations of this review.

 Cannot yet answer/conclude whether the interaction between S. mutans and Veillonellae sp on dental caries progression has a positive or negative impact

Are the rationale for, and objectives of, the Systematic Review clearly stated?

Yes

Is the statistical analysis and its interpretation appropriate?

I cannot comment. A qualified statistician is required.

If this is a Living Systematic Review, is the ‘living’ method appropriate and is the search schedule clearly defined and justified? (‘Living Systematic Review’ or a variation of this term should be included in the title.)

Yes

Are sufficient details of the methods and analysis provided to allow replication by others?

Partly

Are the conclusions drawn adequately supported by the results presented in the review?

Partly

Reviewer Expertise:

Oral Microbiology, Oral Biology

We confirm that we have read this submission and believe that we have an appropriate level of expertise to confirm that it is of an acceptable scientific standard, however we have significant reservations, as outlined above.

# Reviewer report: Reviewer response for version 1

**DOI:** 10.5256/f1000research.171233.r350938

**Published:** 2025-01-08

**Authors:** Fidya Fidya

**Affiliations:** 1 Universitas Brawijaya, Malang, East Java, Indonesia

This study uses a systematic review to investigate the interaction between S. mutans and Veillonella spp. on caries development. The result revealed important aspects of the relationship between *S. mutans* and *Veillonellae spp.* in dental caries.

 One significant finding is that *Veillonellae spp.* can affect the acid production capacity of *S. mutan*, potentially influencing its cariogenic potential. This systematic review was accorded to the guideline of the Preferred Reporting Items for Systematic Reviews and Meta-Analyses provided to allow replication by others.

 This review highlights the emerging evidence on the interaction between *S. mutans* and *Veillonellae spp.* in dental caries. The findings suggest that *Veillonellae spp.* can modulate the acid production and substrate competition of S. mutans, potentially influencing the cariogenic process. The conclusion supports the result presented in the review.

Are the rationale for, and objectives of, the Systematic Review clearly stated?

Yes

Is the statistical analysis and its interpretation appropriate?

Not applicable

If this is a Living Systematic Review, is the ‘living’ method appropriate and is the search schedule clearly defined and justified? (‘Living Systematic Review’ or a variation of this term should be included in the title.)

Not applicable

Are sufficient details of the methods and analysis provided to allow replication by others?

Yes

Are the conclusions drawn adequately supported by the results presented in the review?

Yes

Reviewer Expertise:

Oral Biology, Anatomy,  Histology, Cell biology

I confirm that I have read this submission and believe that I have an appropriate level of expertise to confirm that it is of an acceptable scientific standard.

# Response

**Published:** 2025-01-12

**Authors:** Hendrik Setia Budi

**Affiliations:** Airlangga University, Indonesia

Thank you for the review and acknowledgement.

# Reviewer report: Reviewer response for version 1

**DOI:** 10.5256/f1000research.171233.r342753

**Published:** 2024-12-30

**Authors:** Zahreni Hamzah, Yani Corvianindya Rahayu

**Affiliations:** 1 Department of Biomedical Science, Faculty of Dentistry, University of Jember, Jember, Indonesia; 2 University of Jember, Jember, Indonesia

This article is very good and interesting for fellow dentists to know. Based on the literature used, it is proven that S. Mutans and Veillonella spp. have an interrelated role with each other in maintaining homeostasis in cases of dental caries. This article is very worthy for indexing. However, I as the reviewer stated that this article only needs a little improvement. 

 The revisions that need to be made are as follows:

 1.  There needs to be consistency in writing S. Mutans, for example: the writing of S. Mutans is written as S. Mutams.

 2.  There needs to be consistency in the writing of Veillonella, it should be written “Veillonella spp. Please check  in this article.

Are the rationale for, and objectives of, the Systematic Review clearly stated?

Yes

Is the statistical analysis and its interpretation appropriate?

Yes

If this is a Living Systematic Review, is the ‘living’ method appropriate and is the search schedule clearly defined and justified? (‘Living Systematic Review’ or a variation of this term should be included in the title.)

Yes

Are sufficient details of the methods and analysis provided to allow replication by others?

Yes

Are the conclusions drawn adequately supported by the results presented in the review?

Yes

Reviewer Expertise:

Biomedical Oralmaxillofacial Pathology, Oral Biology, Gerodontology

We confirm that we have read this submission and believe that we have an appropriate level of expertise to confirm that it is of an acceptable scientific standard, however we have significant reservations, as outlined above.

# Response

**Published:** 2025-01-12

**Authors:** Hendrik Setia Budi

**Affiliations:** Airlangga University, Indonesia

The authors express gratitude to the reviewer for the favorable feedback and for emphasizing the significance of this study for the dental community. We acknowledge the recommendations for enhancement and have responded to them as outlined below:

 We have conducted a thorough review of the manuscript and rectified all inconsistencies in the writing of S. mutans. All instances now uniformly adhere to the correct format. S. mutans.

 Consistency in Writing Veillonella spp.: We have maintained uniformity in the representation of Veillonella spp. throughout the manuscript. All inconsistencies have been addressed.

 We anticipate that these revisions will fulfill your expectations and improve the manuscript's quality. I appreciate your insightful comments and suggestions.

# Reviewer report: Reviewer response for version 1

**DOI:** 10.5256/f1000research.171233.r350944

**Published:** 2024-12-28

**Authors:** Yifei Zhang

**Affiliations:** 1 Peking University School and Hospital of Stomatology, Beijing, China

The main defect of this review is that the author only included clinical research literature and can only draw relevant conclusions, but could not draw causal conclusions, which need robust experimental data.

 Besides, there are some misunderstandings about basic concepts, which may affect the quality of the article. For example:

 “ *The main oral bacteria are gram-positive and gram-negative, followed by aerobic and anaerobic*”  

 Gram-stain reflects the cell wall structure, while aerobic/anaerobic reflects the growth characteristics.

*Polymicrobial community in oral biofilms include Streptococcus, Actinomyces,*

*Lactobacillus, Veillonellae, Neisseria, and Eubacterium.5 This biofilm can thrive in homeostasis with a human host but is also the cause of the two most common human diseases, dental caries and periodontitis*

 Those bacteria are not the main members that causing periodontitis.

*Streptococcus mutans (S. mutans) is a normal flora in the oral cavity, including gram-positive facultative anaerobes.*

 S. mutans is a species.

*In certain circumstances, these bacteria can turn into pathogens due to predisposing factors, namely oral hygiene.*

 Oral hygiene is a predisposing factor? What does it mean?

Are the rationale for, and objectives of, the Systematic Review clearly stated?

Partly

Is the statistical analysis and its interpretation appropriate?

I cannot comment. A qualified statistician is required.

If this is a Living Systematic Review, is the ‘living’ method appropriate and is the search schedule clearly defined and justified? (‘Living Systematic Review’ or a variation of this term should be included in the title.)

Not applicable

Are sufficient details of the methods and analysis provided to allow replication by others?

Partly

Are the conclusions drawn adequately supported by the results presented in the review?

No

Reviewer Expertise:

Oral microbiology

I confirm that I have read this submission and believe that I have an appropriate level of expertise to state that I do not consider it to be of an acceptable scientific standard, for reasons outlined above.

# Response

**Published:** 2025-01-12

**Authors:** Hendrik Setia Budi

**Affiliations:** Airlangga University, Indonesia

We appreciate the reviewer's insightful comments and constructive feedback. We will now address the specific concerns raised.

 The incorporation of clinical research literature and the constraints in establishing causal conclusions. The reliance on clinical research literature presents limitations that restrict the capacity to draw causal conclusions. The manuscript explicitly outlines this limitation. The revised text includes a discussion highlighting the necessity of robust experimental data to support causal inference.

 Misinterpretation of Fundamental Principles:

 Gram stain and growth characteristics: Thank you for highlighting the inaccuracies in the phrasing related to gram-positive/negative and aerobic/anaerobic bacteria. The statement has been revised to clarify that gram-stain indicates cell wall structure, whereas aerobic/anaerobic pertains to growth characteristics. The revised text states: "Oral bacteria encompass both gram-positive and gram-negative species exhibiting varied growth characteristics, including aerobic and anaerobic organisms."

 The composition of the polymicrobial community in oral biofilms and its association with periodontitis has been clarified, and we acknowledge this correction. The text has been revised to accurately state: "The polymicrobial community in oral biofilms, comprising Streptococcus, Actinomyces, Lactobacillus, Veillonella, Neisseria, and Eubacterium, exists in homeostasis with the human host." Specific bacteria, including Porphyromonas gingivalis, Tannerella forsythia, and Treponema denticola, exhibit a strong association with periodontitis.

 Clarification regarding Streptococcus mutans (S. mutans): The characterization of S. mutans as "normal flora" has been refined to prevent misconceptions. The updated text specifies that S. mutans is a gram-positive facultative anaerobe typically located in the oral cavity. Under specific conditions, including inadequate oral hygiene, S. mutans may play a role in the formation of dental caries.

 Oral hygiene as a predisposing factor.

 The statement "oral hygiene is a predisposing factor" is indeed ambiguous and necessitates further clarification. The updated text clarifies: "Inadequate oral hygiene fosters conditions that promote the proliferation of harmful bacteria, increasing the risk of oral diseases like caries and periodontitis."

 The revisions aim to address the raised concerns and enhance the clarity and quality of the manuscript.
